# Functional connectivity during orthographic, phonological, and semantic processing of Chinese characters identifies distinct visuospatial and phonosemantic networks

**DOI:** 10.1002/hbm.26075

**Published:** 2022-09-12

**Authors:** Chun Yin Liu, Ran Tao, Lang Qin, Stephen Matthews, Wai Ting Siok

**Affiliations:** ^1^ Department of Linguistics The University of Hong Kong Hong Kong SAR China; ^2^ Research Centre for Language, Cognition, and Neuroscience, Department of Chinese and Bilingual Studies The Hong Kong Polytechnic University Hong Kong SAR China; ^3^ Center for MRI Research, Academy for Advanced Interdisciplinary Studies Peking University Beijing China

**Keywords:** Chinese reading, dorsolateral prefrontal cortex, fMRI, functional connectivity, reading constituents

## Abstract

While neuroimaging studies have identified brain regions associated with single word reading, its three constituents, namely, orthography, phonology, and meaning, and the functional connectivity of their networks remain underexplored. This study examined the neurocognitive underpinnings of these neural activations and functional connectivity of the identified brain regions using a within‐subject design. Thirty‐one native Mandarin speakers performed orthographic, phonological, and semantic judgment tasks during functional magnetic resonance imaging. The results indicated that the three processes shared a core network consisting of a large region in the left prefrontal cortex, fusiform gyrus, and medial superior frontal gyrus but not the superior temporal gyrus. Orthographic processing more strongly recruited the left dorsolateral prefrontal cortex, left superior parietal lobule and bilateral fusiform gyri; semantic processing more strongly recruited the left inferior frontal gyrus and left middle temporal gyrus, whereas phonological processing more strongly activated the dorsal part of the precentral gyrus. Functional connectivity analysis identified a posterior visuospatial network and a frontal phonosemantic network interfaced by the left middle frontal gyrus. We conclude that reading Chinese recruits cognitive resources that correspond to basic task demands with unique features best explained in connection with the individual reading subprocesses.

## Graphical abstract

This study examines the neurocognitive underpinnings of orthographic, phonological, and semantic processing and functional connectivity of the identified brain regions using a within‐subject design. The results indicated that the three processes were supported by a large common network but had different connectivity patterns.

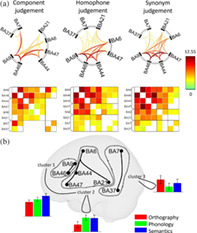

## 1 INTRODUCTION

Neuroimaging studies in the past three decades have well identified a left‐lateralized brain network for reading, comprising the inferior frontal (or Broca's area) and occipitotemporal and temporoparietal regions (which coincide with Wernicke's area) (see Price, 2012 for a review). This neural network for reading overlaps largely with the spoken language network and is argued to be culturally universal (e.g., Feng et al., 2020; Nakamura et al., 2012; Rueckl et al., 2015), although some studies have shown that the neural mechanisms underlying reading and reading impairments may be shaped by language‐specific factors (Bolger et al., 2005; Paulesu et al., 2000; Siok et al., 2008; Siok et al., 2004; Siok et al., 2009; Tan et al., 2005; Tan et al., 2003;). For example, Chinese readers seldom use the left temporoparietal region, particularly the left superior temporal gyrus (STG), but more robustly recruit the left middle frontal gyrus (LMFG), left superior parietal lobule (SPL), bilateral fusiform gyri (FFG), and bilateral lingual gyri and cuneus to process orthophonological information (Tan et al., 2005; Wu et al., 2012). These divergent findings may arise due to variability in the tasks used that tap into different aspects of reading, such as visuo‐orthographic, phonological, and semantic processing. Indeed, studies reporting universal reading networks used implicit reading tasks that measured automatic reading processing (Krafnick et al., 2016) or simple decision tasks that involved language‐general semantic processing (Dehaene & Cohen, 2011; Hu et al., 2010; Nakamura et al., 2012; Rueckl et al., 2015), whereas studies claiming cultural‐specific reading networks examined phonological activation during visual word identification (Siok et al., 2004; Siok et al., 2008; Tan et al., 2005). Since reading is a complex phenomenon involving multiple cognitive and linguistic processes, these inconsistent findings highlight the need to delimit the neural networks underlying various aspects of reading processing. The purpose of this study was to examine the neurocognitive bases of the three constituents of reading (i.e., orthography, phonology, and meaning) in Chinese.

Spoken language uses sound to convey meaning. Thus, language acquisition involves the mapping of sound to meaning, rendering the brain networks for phonological and semantic processing in young children highly overlapped and connected (e.g., Mathur et al., 2020; Weiss et al., 2018). It has long been reported that languages used across the world are commonly supported by a left‐lateralized language network comprising Broca's and Wernicke's areas. When children start learning to read, they have to associate the newly acquired written symbols with the already existing phonological and semantic constituents (Perfetti et al., 2005), resulting in the wiring and rewiring of the visual cortex with the spoken language network (Dehaene, 2009; Orton, 1925; Schlaggar & McCandliss, 2007; Skeide et al., 2017). Here, the learning process and the resultant neural mechanisms for reading may be dependent on the visual configuration of the written symbols used and the manner of print‐to‐sound mapping. In alphabetic scripts, a grapheme (the smallest written unit, either a letter or letter cluster) represents a phoneme (the smallest contrastive speech unit) in a quasi‐systematic manner, with word meanings derived through the mediation of letter‐sound correspondence. Thus, processing alphabetic scripts requires the dorsal circuit, including the left temporoparietal regions, for rule‐based phonological processing and the ventral circuit, including the occipitotemporal regions, for memory‐based word recognition (Pugh et al., 2001).

The Chinese writing system does not represent individual phonemes of a word. Each Chinese character encodes a morphosyllabic unit, and its pronunciation is usually underdetermined from the stroke patterns or character components. Accordingly, character pronunciation must be memorized by rote and cannot be deduced by recourse to a prelexical, rule‐based phonological computation process that is available in alphabetic systems. Thus, not surprisingly, the left temporoparietal region, particularly the STG, is seldom observed to be involved in Chinese orthography‐to‐phonology processing (Tan et al., 2005). This type of memory‐based phonological process, known as addressed phonology, is also available in alphabetic systems for reading words with high frequency or irregular spelling patterns (Coltheart, 2006), and reading written scripts in both systems activates the ventral reading circuit. However, contrary to alphabetic systems, phonology does not mediate meaning access in Chinese character reading, as homophones abound (Tan & Perfetti, 1998), although phonological activation precedes semantics (Tan et al., 1995; Tan et al., 1996) and is obligatory during meaning access (Spinks et al., 2000). This one‐to‐many relationship between phonology and meaning may weaken the connection between them, whereas the mostly one‐to‐one mapping between orthography and phonology may make these two processes more closely associated (Perfetti & Tan, 1998; Tan & Perfetti, 1998). Based on the above analysis, the reading network for Chinese reading may involve two possible patterns of relationships among orthography, phonology, and meaning: phonology and meaning are more integrated because this relationship is shaped by spoken language experience, or phonology and orthography are more associated because form‐form relations are more reliable than form‐meaning relations (Perfetti & Tan, 1998). A second purpose of this study was to examine these two possibilities. We examined the relationships among the three networks by depicting the relative strength in activation and connectivity among specialized regions of interest (ROIs).

One of the often reported peculiarities of neural activation during Chinese reading is activation in the left dorsolateral frontal regions. This region, including the LMFG (BA 9/46), has been observed to engage in studies examining various types of Chinese reading‐related processing, such as phonological (e.g., Chen et al., 2008; Kim et al., 2016; Klein et al., 2001; Kuo et al., 2004; Siok et al., 2003; Siok et al., 2004; Siok et al., 2008; Tan et al., 2003; Tan, Feng, et al., 2001), orthographic (Cao et al., 2013; Kuo et al., 2004), semantic (Booth et al., 2006; Chee et al., 2000; Ding et al., 2003; Siok et al., 2004; Tan, Liu, et al., 2001; Wu et al., 2012) and syntactic (Luke et al., 2002) processing, as well as text reading (Zhou et al., 2016) and writing (Cao et al., 2013). Although the LMFG has also been found to be involved in alphabetic reading (Feng et al., 2020; Hu et al., 2010; Murphy et al., 2019), many studies do not report LMFG activation (e.g., Binder et al., 1997; Houdé et al., 2010; Paulesu et al., 2014; Turkeltaub et al., 2002), which suggests that the LMFG is not as robustly recruited in alphabetic reading as it is in Chinese reading. Indeed, the LMFG likely serves domain‐general functions and has been shown to play an important role in higher‐order cognitive functions such as working memory (Curtis & D'Esposito, 2003; D'Esposito & Postle, 2015; Kessels et al., 2000), executive control, and attention (Ihnen et al., 2015; MacDonald, 2000; Petersen & Posner, 2012). As Chinese reading requires intensive visuospatial analysis and rote memorization of the print‐sound‐meaning mapping of characters, the LMFG may be recruited to serve or coordinate these functions. A third objective of this study was to scrutinize the cognitive functions served by the left middle frontal region that are pertinent to Chinese reading.

We addressed two questions. The first question concerns the cortical networks underlying individual types of processing in Chinese reading: Are there specialized and distinct regions for orthographic, phonological, and semantic processing? Studies comparing these processes suggested that the left inferior frontal gyrus (IFG), FFG, and SPL are the loci for task‐specific activations (Booth et al., 2006; Guo & Burgund, 2010; Kuo et al., 2004; Liu et al., 2006; Liu et al., 2009). However, these studies either compared only two of the processes (Booth et al., 2006; Cao et al., 2009; Kuo et al., 2004), focused on specific ROIs (Guo & Burgund, 2010) or did not fully report on all possible comparisons (Liu et al., 2006). To fully understand the functional specialization of the three networks, a more powerful yet stringent direct comparison among the three processes using whole‐brain analysis is required. In this study, we utilized a within‐subject design for the between‐task comparisons. As no studies to date have attempted a connectivity‐based comparison of the three processes, we also probed into functional connectivity among the orthographic, phonological, and semantic processing networks in Chinese speakers. Next, we asked how the three networks are interrelated among one another. In alphabetic languages, as the pronunciation of a word can be derived from the spelling through grapheme‐to‐phoneme conversion (GPC), orthographic and phonological processing cannot be fully disentangled. This suggests that these two networks should be similar. Nevertheless, previous studies have demonstrated segregation of the two networks. Phonological processing of alphabetic languages shows activation in the left supramarginal gyrus (SMG), STG, and IFG (e.g., Booth et al., 2002; Paulesu et al., 1996; Pugh et al., 2001), while orthographic processing (usually in terms of spelling judgment) elicits activation in the bilateral FFG and SPL (Booth et al., 2002; Cohen et al., 2000; Petersen et al., 1990; Tagamets et al., 2000). Similarly, phonological and semantic networks have been found to be distinct in the left IFG (Poldrack et al., 1999), middle temporal cortex, SMG, and precentral gyrus (Price et al., 1997). The above descriptions provide two possible types of relationships between the neural networks of the subcomponents of Chinese reading, and this study determined whether Chinese character reading follows one of those patterns.

Component, homophone, and synonym judgment tasks were used to probe into orthographic, phonological, and semantic processing, respectively. The phonological and semantic tasks were paired with a visual‐complexity‐matched font‐size judgment baseline task to account for activations arising from orthographic analysis. The orthographic task was paired with a line pattern judgment baseline task to extract the activation for finer visuo‐orthographic analysis of the internal structure of Chinese characters. The activation and connectivity profiles of the three processes were analyzed by whole‐brain, ROI, and ROI‐to‐ROI connectivity approaches. It was predicted that regions specialized for a particular process will exhibit stronger activation levels and interconnections among themselves, hence forming a functional unit. Commonly activated regions that are not specialized for any particular task, probably the LMFG, may act as an integration hub that connects separate processes in Chinese reading.

## 2 MATERIALS AND METHODS

### 2.1 Participants

Here, 31 native Mandarin speakers were recruited at Peking University (21 males and 10 females; mean age = 20.9 years, SD = 1.68 years). All participants were undergraduate or graduate students, had normal or corrected‐to‐normal vision and were free of any neurological or psychiatric disorders. All participants except one were right‐handed as judged by the Edinburgh handedness inventory (Oldfield, 1971). The one exception obtained a score of +40, which is considered marginal between ambidextrous and right‐handed. The activation maps of this participant showed a typical left‐lateralized pattern and were included in the group analysis. Written informed consent was obtained from each participant, and the study was approved by the Human Research Ethics Committee at the University of Hong Kong. All scanning protocols were approved by the Peking University Institutional Review Boards.

### 2.2 Materials

**FIGURE 1:**
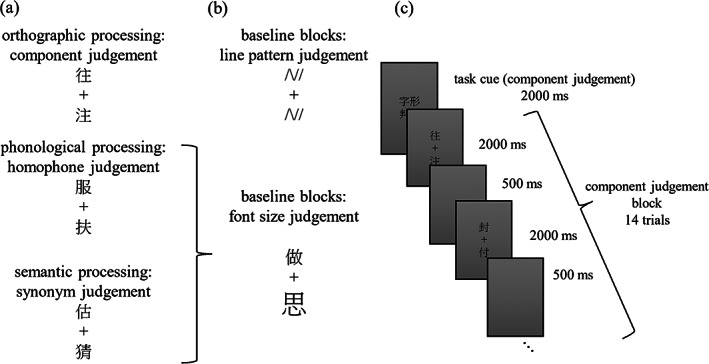
Experimental paradigms used during functional magnetic resonance imaging (fMRI) scans. (a) Sample trials for the orthographic, phonological, and semantic processing tasks. (b) Sample trials for the baseline tasks. (c) The organization and time course of a task block

A component judgment task, a homophone decision task, and a synonym judgment task were used to probe the orthographic, phonological, and semantic processing of Chinese character reading, respectively, in different runs (Figure 1a). For each task, 28 pairs of characters with shared components, homophones, or synonyms were displayed, and the participants were asked to judge if the two characters shared the same component, pronunciation or meaning in the orthographic, phonological, and semantic tasks, respectively. An extra 28 pairs of characters were included in each task as fillers to balance the number of correct yes and no responses. The frequency and stroke number of the characters were matched across the three tasks (Table 1). In each trial, a pair of characters were simultaneously presented above and below a fixation cross for 2 s, followed by a 500‐ms blank. Trials were organized into blocks of 14 and were presented in the same pseudorandomized order for each participant. In the orthographic task, component judgment blocks were alternated with line pattern judgment blocks (Figure 1b) in which the participants judged whether two line patterns were the same. In the phonological and semantic tasks, experimental blocks alternated with font‐size judgment blocks in which the participants judged if the two characters had the same size. A task cue of 2 s appeared at the beginning of each block (Figure 1c). The three tasks were performed during three separate scans, which lasted for 310 s each.

**TABLE 1:** The mean number of strokes and frequency of the Chinese characters used in each task. SD, standard deviation

Task	Number of strokes		Frequency (per million)	
	Mean	SD	Mean	SD
Orthography	9.63	2.49	321.85	577.97
Phonology	8.91	2.63	347.69	787.96
Semantics	8.96	2.38	294.19	629.87

### 2.3 Procedures

#### 2.3.1 Stimuli presentation and behavioral data acquisition

Stimuli presentation and response data‐logging were interfaced by E‐prime 2.0. The participants lay in a supine position while seeing stimuli back‐projected onto a screen through a mirror mounted in a 64‐channel head coil. The participants indicated a “yes” response by pressing a button with their right index finger and a “no” response with their right middle finger. The participants were familiarized with all the tasks using materials that did not overlap with the experimental stimuli before the scans. The order of performing the three tasks was counterbalanced among the participants.

#### 2.3.2 Image acquisition

Functional magnetic resonance imaging (fMRI) data were acquired with a 3 T Siemens MAGNETOM Prisma scanner in the Centre for MRI Research at Peking University. High‐resolution (0.5 × 0.5 × 1 mm^3^) T1‐weighted anatomical brain images were acquired using a three‐dimensional magnetization‐prepared rapid acquisition gradient‐echo sequence (repetition time [TR] = 2530 ms, echo time [TE] = 2.98 ms, inversion time = 1100 ms, flip angle [FA] = 7°, number of slices = 192), while fMRI data were collected using a T2*‐weighted gradient echo‐planar imaging sequence (TR = 2000 ms, TE = 30 ms, FA = 90°, number of slices = 33, interleaved, slice thickness = 3.5 mm, gap = 0.7 mm, matrix = 64 × 64, in‐plane resolution = 3.5 × 3.5 mm^2^). Each scan contained 155 volumes.

### 2.4 fMRI data analysis

#### 2.4.1 Preprocessing

Image preprocessing was performed with the default preprocessing pipeline in the CONN toolbox (Whitfield‐Gabrieli & Nieto‐Castanon, 2012) in MATLAB 2019b and SPM12. Functional images were first motion corrected and time sliced. Outlier scans were then identified by the ART‐based outlier detection procedure, where images with framewise displacement greater than 0.9 mm or with global BOLD signal changes greater than 5 SDs were identified for scrubbing (Nieto‐Castanon, 2020). The functional images were then segmented and normalized to the ICBM standard template in MNI space at a resolution of 2 × 2 × 2 mm, and smoothed with an isotropic 8‐mm full‐width at half‐maximum Gaussian kernel. Participants with head motion exceeding 1 mm within a single run were excluded. In total, twenty‐two scans from six participants were identified as motion‐ or signal‐change outliers; the maximum number of scans discarded within the same run was 4. The maximum head motion of all participants was less than 1 mm; thus, no participants were excluded due to excessive head motion.

#### 2.4.2 Common and task‐specific activations

Preprocessed images of individual participants were submitted to a first‐level analysis using a general linear model. The experimental task blocks and the baseline blocks were modeled by two separate regressors that were convolved with a canonical hemodynamic response function. The six head motion parameters estimated during the motion correction step, independent regressors for the scrubbed scans and the global BOLD signal change were included to regress out motion artifacts and to remove the effects of motion and signal change spikes, respectively. The time series data were high‐pass filtered at 128 s and modeled by FAST (Corbin et al., 2018). The group activation t‐maps for each task were then obtained by performing second‐level random‐effect analysis on the contrast images between the task and baseline blocks for each participant. To determine the regions common to the three processes, a conjunction analysis at the second level was conducted. The effects of the three tasks were tested against the conjunction null (Nichols et al., 2005). To discover the regions specific to individual tasks, the t‐contrast maps obtained from the three tasks were entered into a one‐way within‐subject ANOVA with unequal variance. An uncorrected *p* value of .001 and a familywise error rate (FWE)‐corrected *p* value of .05 were applied to the voxelwise and cluster‐level statistical significance for all contrasts.

#### 2.4.3 ROI analysis

To further illustrate how commonly reported and debated regions for Chinese reading are involved in specific processing tasks, the contrast estimates of the three tasks were extracted from the left frontal, parietal, temporal and bilateral occipitotemporal regions using rfxplot (Gläscher, 2009). Three axes were defined for the left frontal region: a superior axis from MNI coordinates (−48, 0, 50) to (−48, 48, 14); a middle axis from (−48, 0, 32) to (−48, 48, −8); and an inferior axis from (−46, −4, 14) to (−46, 36, −14) (Figure 3a). The axes in the bilateral FFG were defined from (±43, −42, −16) to (±39, −88, −10) (Figure 3b). Five evenly spaced 6‐mm spherical ROIs were defined along each axis, and the average contrast estimates of the three tasks were extracted from the ROIs. For the parietal region, (−28, −54, 38) at the left SPL was chosen from the F‐contrast map. Finally, (−56, −40, 14) at the left superior temporal sulcus (STS) was chosen to test whether it was associated with any phonological effects. A posterior and inferior region in the middle temporal gyrus (MTG) at (−62, −42, 0), which is well documented for its role in semantic processing (e.g., Price, 2012), was added for comparison. Repeated‐measures ANOVA was performed on the contrast estimates of each ROI with task as the within‐subject factor using the lme4 package in R (Bates et al., 2015). Post hoc pairwise comparisons between the three tasks were performed with the emmeans package (formerly the lsmeans package; Lenth, 2016). To adjust for multiple comparisons among the 28 ROIs and the post hoc tests, a Bonferroni‐corrected *p* value of .05 was applied.

#### 2.4.4 Connectivity analysis

To further differentiate the neural networks for orthographic, phonological, and semantic processing in Chinese character reading and pinpoint the functional specialization of the LMFG, a functional connectivity analysis was conducted in the CONN toolbox (Whitfield‐Gabrieli & Nieto‐Castanon, 2012). We focused on major left ROIs that are commonly reported in Chinese reading and selected eight coordinates from Wu et al. (2012): BA 9 (−48, 14, 32); BA 46 (−48, 26, 16); BA 44 (−52, 12, 16); BA 47 (−46, 28, −4); BA 21 (−58, −44, 0); BA 6 (−46, 0, 54); BA 7 (−26, −66, 54); and BA 37 (−44, −60, −14). For each ROI, the average signals of a 6‐mm sphere were extracted from the preprocessed functional images. The resultant time series were denoised with CONN's default pipeline, which included scrubbing, removal of task‐based, structural‐based and motion confounds, temporal filtering, and linear detrending. Functional connectivity during the orthographic, phonological, and semantic task blocks between each pair of ROIs was calculated with Pearson correlation coefficients at the first level. Correlation coefficients were Fisher‐transformed into z scores and tested against zero at the second level. Second‐level significance was tested by cluster‐based functional network connectivity multivariate parametric statistics, thresholded at a cluster level of *p* < .05, with false discovery rate (FDR) correction (Jafri et al., 2008). The ROI clusters were sorted by hierarchical clustering of their functional similarity. We also tested for connectivity differences among the three tasks at the cluster and ROI‐to‐ROI connection levels.

## 3 RESULTS

### 3.1 Behavioral results

The accuracy rates and reaction times for the three tasks are reported in Table 2. A task‐by‐block‐type (3 × 2) factorial ANOVA was performed on the accuracy rates and reaction time in R. The main effects and interaction effect with the accuracy data were not significant (task: *F*(2,180) = 1.45, *p* = .238; block type: *F*(1,180) = 0.646, *p* = .423; interaction: *F*(2,180) = 0.700, *p* = .498). With the reaction times, there was a strong main effect of block type (*F*(1,180) = 170, *p* = 8.86 × 10^−28^), with the reaction times in the baseline blocks being shorter than those in the reading task blocks. The main effect of Task and the interaction term were not significant (task: *F*(2,180) = 2.26, *p* = .107; interaction: *F*(2,180) = 0.195, *p* = .823). The absence of the main effect of task and task‐by‐block‐type interaction indicated that the three reading tasks and the three baseline tasks were equally demanding, which justified across‐scan comparisons.

**TABLE 2:** Behavioral data from the three fMRI tasks. SD, standard deviation

Task	Accuracy				Reaction time (ms)			
	Reading task blocks		Baseline blocks		Reading task blocks		Baseline blocks	
	Mean	SD	Mean	SD	Mean	SD	Mean	SD
Orthography	0.932	0.052	0.955	0.036	1067.998	114.499	863.487	110.348
Phonology	0.925	0.083	0.925	0.072	1070.403	117.243	840.774	116.253
Semantics	0.930	0.065	0.929	0.056	1031.385	101.230	817.257	106.818

Abbreviation: fMRI, functional magnetic resonance imaging.

### 3.2 fMRI contrasts

Due to the imbalance of sex, we first conducted an independent‐sample *t* test between males and females to check if there were sex effects. No clusters survived the task contrast comparison (voxel‐level: *p* = .001, uncorrected; cluster‐level: *p* = .05, FWE corrected). fMRI data for all participants were pooled together for subsequent analyses.

**FIGURE 2:**
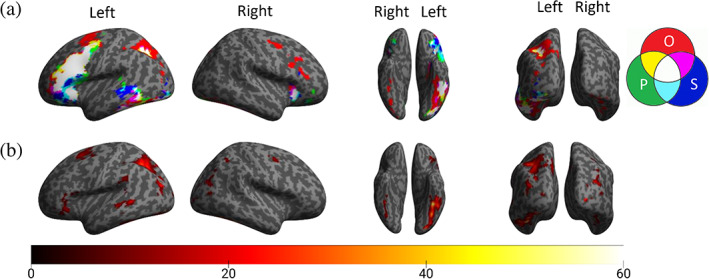
(a) Significantly activated regions in the orthographic, phonological, and semantic tasks and their intersection rendered on an inflated brain. The Venn diagram illustrates the color codes for the three activations. O: orthographic activation; P: phonological activation; S: semantic activation. (b) The F‐contrast map from within‐subject ANOVA shows regions with significantly different activation levels among the three tasks. Both (a) and (b) are thresholded at voxel‐level *p* < .001, uncorrected, and cluster‐level *p* < .05, familywise error rate (FWE) corrected

Activation patterns and peak coordinates in individual tasks are reported in Figure 2a and the Supplementary materials (Tables [Link], [Link]). The results of the conjunction analysis and within‐subject F contrasts are reported in Figure 2b and Table 3. Here, we focus on the commonalities and differences among the three tasks. Consistent with previous meta‐analyses, the conjunction analysis revealed that orthographic, phonological, and semantic processing of Chinese characters were supported by a common network including (i) a large portion of the left frontal region extending ventrally and rostrally from the precentral gyrus (BA 6) through the MFG (BA 9/44) to the IFG (BA 47), (ii) the bilateral insula (BA 13), (iii) the left FFG (BA 37), (iv) the left medial superior frontal gyrus (BA 8), and (v) the right cerebellum. Within‐subject ANOVA revealed that differences in activation were located in the left IFG (BA 44/45/47), precentral gyrus (BA 6), bilateral FFG (BA 37), bilateral SPL/precuneus (BA 7), bilateral angular gyrus/SMG (BA 39/40), left MTG (BA 21), and bilateral middle/inferior occipital gyri (M/IOG) (BA 18/19). Pairwise comparisons of activation level in selected sites will be discussed in the ROI analysis in the next section.

**TABLE 3:** MNI coordinates of the peaks found in the conjunction analysis and within‐subject ANOVA. The *p* values are uncorrected. The clusters survived a statistical significance of *p* < .05 with FWE correction

Regions		*k*	BA	MNI coordinates			Peak *t*/*F*	*p*
				x	y	z		
*Conjunction analysis (t‐contrast)*								
Left	Middle/inferior frontal gyrus		44	−42	22	22	10.87	4.44 × 10^−16^
			44	−42	12	26	9.84	5.55 × 10^−16^
	Insula	4661	13	−28	22	−2	6.20	8.23 × 10^−09^
	Precentral gyrus	4661	6	−48	4	50	5.51	1.70 × 10^−07^
			6	−40	−2	60	4.43	1.33 × 10^−05^
	Pars orbitalis	4661	47	−34	36	−16	3.78	1.40 × 10^−04^
	Medial superior frontal gyrus	987	8	−2	18	52	6.68	9.77 × 10^−10^
	Inferior temporal gyrus/fusiform gyrus	802	20/37	−48	−36	−24	4.96	1.65 × 10^−06^
			37	−46	−54	−26	4.61	6.54 × 10^−06^
			37	−50	−50	−10	4.43	1.30 × 10^−05^
Right	Insula	277	13	30	26	0	4.89	2.19 × 10^−06^
	Cerebellum	235	‐	8	−72	−36	4.79	3.27 × 10^−06^
*Within‐subject ANOVA (F‐contrast)*								
Left	Fusiform gyrus	2344	37	−38	−40	−28	64.9	1.22 × 10^−15^
			37	−42	−60	−22	46.0	7.81 × 10^−13^
	Cerebellum	2344	‐	−38	−48	−32	53.8	4.20 × 10^−14^
	Superior parietal lobule	2690	7	−28	−54	38	31.3	4.79 × 10^−10^
			7	−6	−68	36	19.0	3.95 × 10^−07^
			7	−24	−76	50	16.3	2.28 × 10^−06^
	Angular gyrus	2690	39	−28	−72	44	19.2	3.67 × 10^−07^
	Middle occipital gyrus	2690	19	−36	−80	20	26.1	6.81 × 10^−09^
	Precentral gyrus	637	6	−24	8	58	20.8	1.34 × 10^−07^
			6	−20	6	50	20.1	2.14 × 10^−07^
			6	−28	−8	48	10.9	9.12 × 10^−05^
	Middle temporal gyrus	333	21	−52	−42	−4	17.7	9.02 × 10^−07^
			21	−62	−42	0	10.8	9.96 × 10^−05^
	Occipital pole	218	18	−18	−86	0	17.5	1.02 × 10^−06^
			18	−10	−92	12	11.3	6.82 × 10^−05^
	Inferior frontal gyrus	505	47	−38	24	−6	16.5	1.99 × 10^−06^
			47	−36	34	−8	15.4	3.88 × 10^−06^
			45	−60	18	16	10.9	9.06 × 10^−05^
			45	−50	26	4	10.8	1.01 × 10^−04^
			44	−52	16	14	9.1	3.44 × 10^−04^
	Supramarginal gyrus	276	40	−62	−36	28	11.9	4.48 × 10^−05^
	Planum temporale	276	22	−52	−40	20	8.9	4.00 × 10^−04^
	Superior temporal gyrus	276	22	−62	−42	12	8.8	4.48 × 10^−04^
	Angular gyrus	276	39	−60	−52	30	8.5	5.76 × 10^−04^
Right	Cerebellum	729	‐	38	−42	−30	31.0	5.70 × 10^−10^
			‐	40	−66	−24	14.5	7.53 × 10^−06^
	Inferior occipital gyrus	373	18	20	−88	4	25.7	8.45 × 10^−09^
	Middle occipital gyrus	261	19	40	−74	20	19.4	3.09 × 10^−07^
			19	38	−76	28	14.5	7.17 × 10^−06^
	Angular gyrus	261	39	42	−72	38	9.7	2.22 × 10^−04^
	Posterior cingulate gyrus	184	31	8	−34	40	17.5	1.05 × 10^−06^
			31	2	−40	52	8.2	7.20 × 10^−04^
	Precentral gyrus	176	6	36	−4	46	14.6	6.97 × 10^−06^
			6	42	0	56	12.3	3.28 × 10^−05^
	Superior parietal lobule	192	7	26	−52	38	12.4	3.02 × 10^−05^

Abbreviations: BA, Brodmann area; FWE, familywise error rate; *k*, cluster size.

### 3.3 Activation profile in the left frontal region, bilateral FFG, and left S/MTG and SPL

**FIGURE 3:**
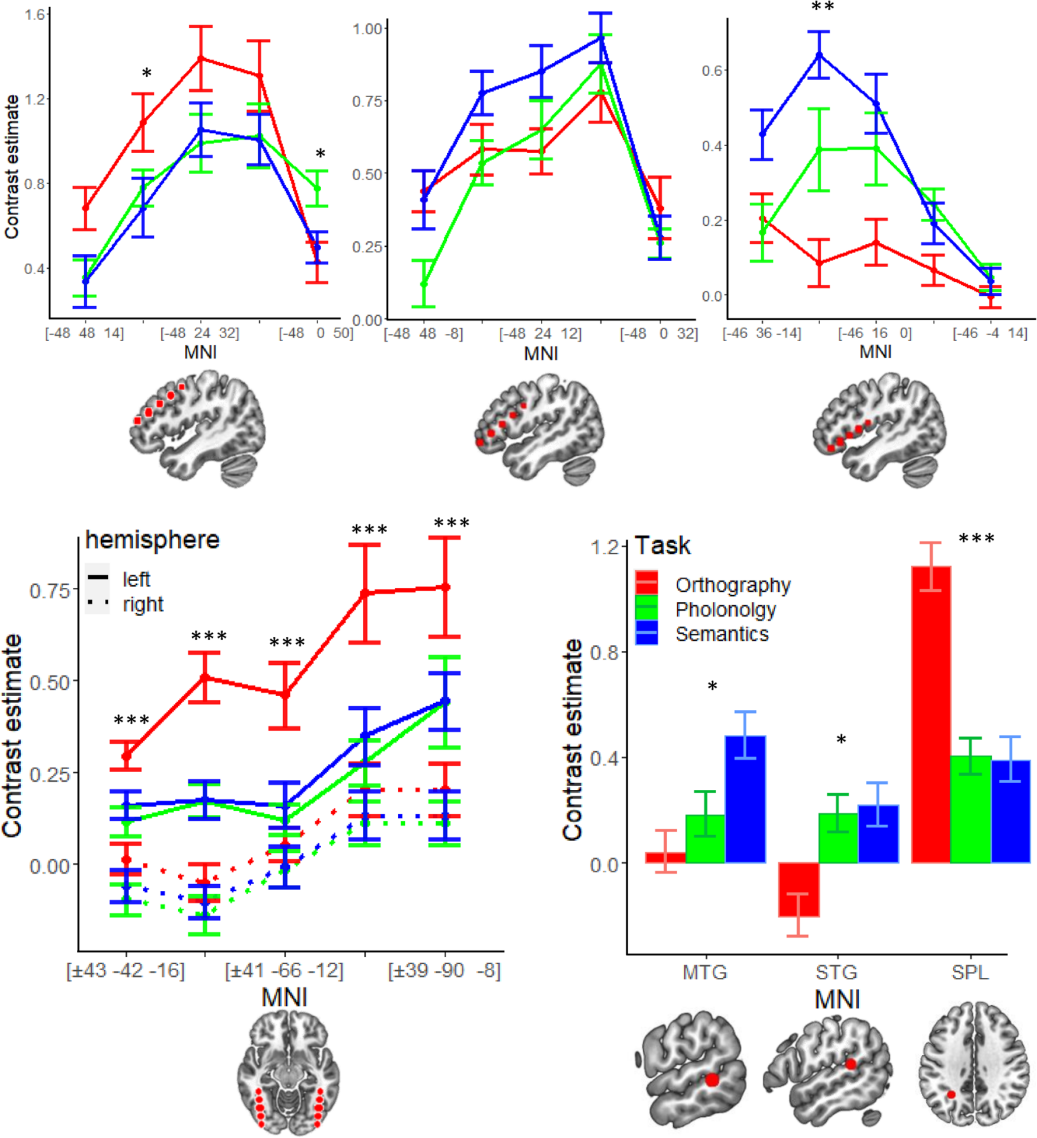
Plots of contrast estimates from the three functional magnetic resonance imaging (fMRI) tasks at selected regions of interest (ROIs). Asterisks indicate a significant difference in activation levels at the ROI. †: *p* < .1; *: *p* < .05; **: *p* < .005; ***: *p* < .0005, Bonferroni corrected

In Figure 3, the contrast estimates from each task were plotted along the five preselected axes (three in the left frontal cortex and two in the bilateral fusiform regions). The corresponding statistics are reported in Table 4. Along the superior axis in the frontal area (sMFG, superior portion of the MFG), contrast estimates from all three tasks showed an inverted‐U shape. At the most posterior and superior coordinate (−48, 0, 50; BA 6), activation during phonological processing was the strongest. Activation in all tasks peaked between (−48, 12, 41; BA 8) and (−48, 24, 32; BA 9) and then decreased. Activation levels during orthographic processing were consistently higher than those during phonological and semantic processing at the second anterior coordinates (i.e., −48, 36, 23; BA 9), while activation levels during phonological processing closely paralleled levels during semantic processing. Along the middle axis (MFG), activation during all tasks also showed an inverted‐U shape. Orthographic processing was the strongest in the most posterior coordinate, although it was not statistically significantly stronger than the other two. Orthographic processing was surpassed by phonological and semantic processing as the coordinates moved rostrally and ventrally. Semantic processing exhibited a trend towards stronger activation compared to phonological processing and orthographic processing at (−48, 36, 2; BA 45) and (−48, 24 12; BA 45) respectively, though the omnibus‐*F* tests were not significant. Along the most ventral axis (IFG), activation during phonological and semantic processing showed an inverted‐U shape, while that of orthographic processing showed a gradually increasing trend when moving in rostral and ventral directions, despite its relatively low activation level. Activations were significantly stronger during both phonological and semantic processing than orthographic processing at (−46, 16, 0; junction of BA 44, 45 and 47). At (−46, 26, −7; BA 47), the pairwise *t* test showed that activation was stronger during semantic processing than both orthographic and phonological processing, though the *F* test was not statistically significant.

**TABLE 4:** Statistics for between‐task comparisons of the contrast estimates across 28 ROIs. All *p* values were Bonferroni corrected

Regions	MNI coordinates			*F*(2,60)	*p*	*t*			*p*		
	x	y	z			O > P	P > S	S > O	O > P	P > S	S > O
sMFG	−48	0	50	8.75	.013*	−3.96	3.15	0.80	6.1 × 10^−04^ **	7.6 × 10^−03^ *	1
	−48	12	41	2.16	1	1.74	0.11	−1.85	.26	1	.21
	−48	24	32	4.2	.55	2.69	−0.41	−2.28	.028*	1	.079^a^
	−48	36	23	7.28	.041*	2.8	0.85	−3.65	.021*	1	1.7 × 10^−03^ **
	−48	48	14	6.08	.11	2.94	0.16	−3.10	.014*	1	9.0 × 10^−03^ *
MFG	−48	0	32	1.45	1	1.58	−0.25	−1.33	.35	1	.56
	−48	12	22	1.17	1	−0.81	−0.72	1.53	1	1	.39
	−48	24	12	4.11	.6	−0.76	−2.02	2.77	1	.14	.022*
	−48	36	2	2.85	1	0.43	−2.25	1.82	1	.085^a^	.22
	−48	48	−8	4.57	.4	2.73	−2.49	−0.25	.025*	.047*	1
IFG	−46	−4	14	1.2	1	−1.48	0.36	1.13	.43	1	.79
	−46	6	7	4.16	.57	−2.8	0.82	1.99	.02*	1	.15
	−46	16	0	5.92	.13	−2.28	−1.09	3.37	.079^a^	.84	3.9 × 10^−03^ **
	−46	26	−7	10.5	3.5 × 10^−03^ **	−2.49	−2.08	4.58	.046*	.13	7.3 × 10^−05^ ***
	−46	36	−14	4.51	.42	0.40	−2.78	2.38	1	.022*	.062^a^
L FFG	−43	−42	−16	74.82	1.4 × 10^−15^ ***	11.29	−1.57	−9.72	5.3 × 10^−16^ ***	.36	1.9 × 10^−13^ ***
	−42	−54	−14	66.52	1.7 × 10^−14^ ***	10.34	−0.74	−9.6	1.8 × 10^−14^ ***	1	3 × 10^−13^ ***
	−41	−66	−12	63.98	3.7 × 10^−14^ ***	10.09	−0.62	−9.47	4.6 × 10^−14^ ***	1	4.8 × 10^−13^ ***
	−40	−78	−10	44.93	3.3 × 10^−11^ ***	8.49	−0.59	−7.90	2.2 × 10^−11^ ***	1	2.2 × 10^−10^ ***
	−39	−90	−8	51.22	3 × 10^−12^ ***	8.71	0.10	−8.82	9 × 10^−12^ ***	1	6.1 × 10^−12^ ***
R FFG	43	−42	−16	25.75	2.4 × 10^−07^ ***	6.94	−1.90	−5.04	9.4 × 10^−09^ ***	.19	1.4 × 10^−05^ ***
	42	−54	−14	30.15	2.4 × 10^−08^ ***	7.50	−2.00	−5.50	1.1 × 10^−09^ ***	.15	2.5 × 10^−06^ ***
	41	−66	−12	20.77	3.9 × 10^−06^ ***	6.03	−1.05	−4.98	3.3 × 10^−07^ ***	.89	1.7 × 10^−05^ ***
	40	−78	−10	20.92	3.6 × 10^−06^ ***	5.93	−0.74	−5.20	4.8 × 10^−07^ ***	1	7.7 × 10^−06^ ***
	39	−90	−8	21.72	2.2 × 10^−06^ ***	5.89	−0.38	−5.51	5.6 × 10^−07^ ***	1	2.4 × 10^−06^ ***
L MTG	−62	−42	0	8.85	.012*	−1.32	−2.80	4.12	.57	.021*	3.5 × 10^−04^ ***
L STG	−56	−40	14	8.92	.011*	−3.50	−0.29	3.80	2.6 × 10^−03^ ***	1	.001**
L SPL	−28	−54	38	30.05	2.5 × 10^−08^ ***	6.66	0.11	−6.77	2.8 × 10^−08^ ***	1	1.9 × 10^−08^ ***

*Note*: O, orthography task; P, phonology task; S, semantic task.

Abbreviations: FFG, fusiform gyri; IFG, inferior frontal gyrus; MFG, middle frontal gyrus; MTG, middle temporal gyrus; ROIs, regions of interest; SPL, superior parietal lobule; STG, superior temporal gyrus.

^a^ *p* < .1.

* *p* < .05.

** *p* < .005.

*** *p* < .0005.

In the bilateral FFG, activation during orthographic processing showed an apparent increasing trend along the anterior–posterior axis. Its activation was significantly stronger than phonological and semantic processing at all coordinates. No comparisons between phonological and semantic processing reached statistical significance. Again, there was a close parallel between the patterns of phonological and semantic processing.

Consistent with previous findings, significantly stronger activation during semantic processing was found in the left MTG (BA 21). In the left STG (BA 22), phonological and semantic activation were significantly stronger than orthographic activation in the pairwise comparison. Finally, activation was significantly stronger during orthographic processing than both phonological and semantic processing in the left SPL (BA 7).

### 3.4 Connectivity analysis

**FIGURE 4:**
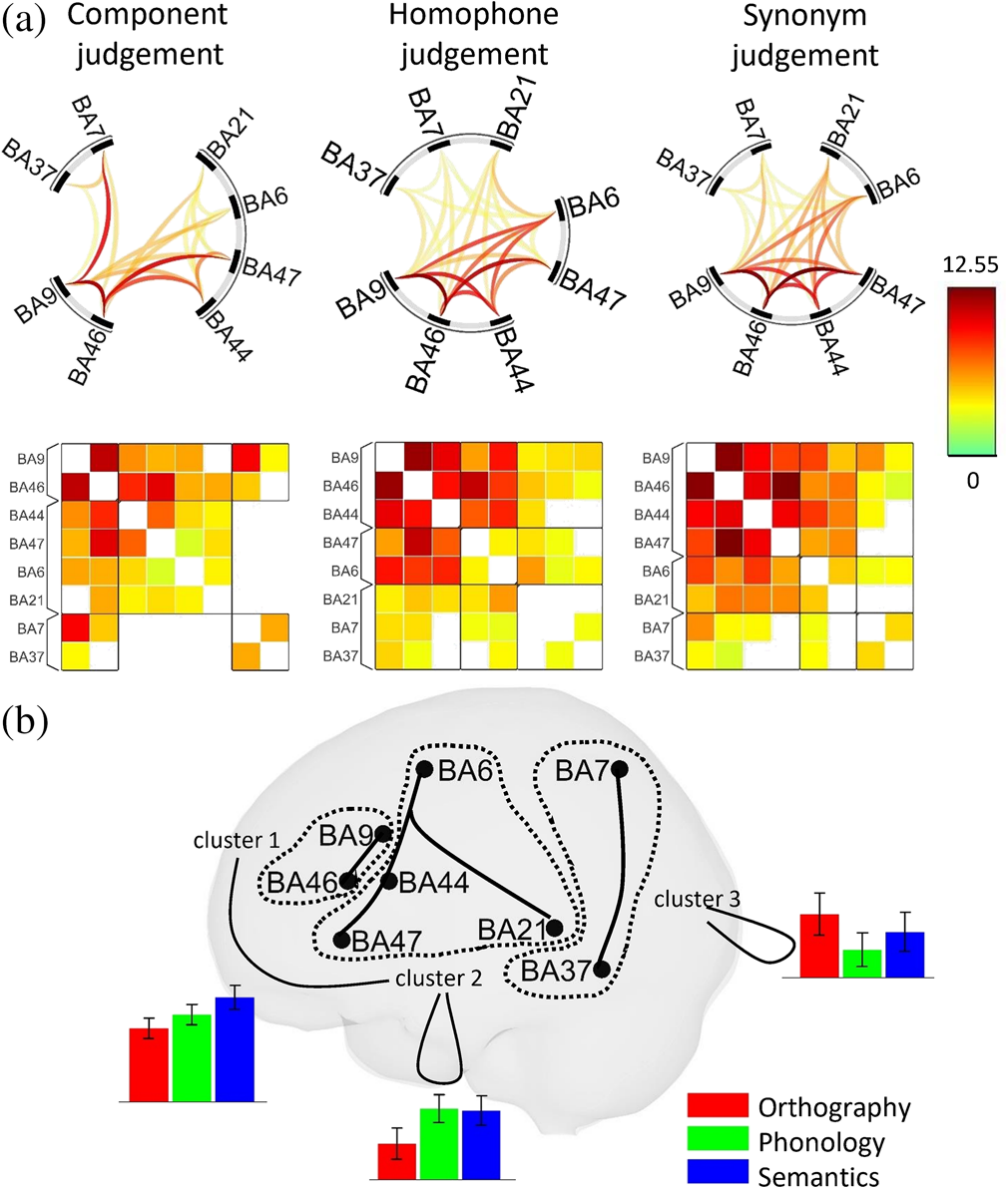
Results of the connectivity analysis. (a) Connectomes and connectivity matrices associated with the three reading tasks. Reported connections survived at a false discovery rate (FDR)‐corrected *p* value of .05. (b). Significant connectivity differences within/between clusters. The color bars show the relative effect sizes of the indicated connectivity (solid black curves)

ROI clusters, connectivity matrices among the eight ROIs during the three reading processes and the differences in connectivity patterns are presented in Figure 4, and the statistics can be found in the Supplementary materials (Table S4). During component judgment, the ROIs were divided into three clusters, which comprised (i) BA 9 and 46; (ii) BA 44, 47, 6, and 21; and (iii) BA 7 and 37. With the exception of the connection between clusters (ii) and (iii), all connections at the cluster level were significant. During homophone judgment, the ROIs were divided into clusters of (i) BA 9, 46, and 44; (ii) BA 47 and 6; and (iii) BA 21, 7, and 37. During synonym judgment, the ROIs were divided into clusters of (i) BA 9, 46, 44, and 47; (ii) BA 6 and 21; and (iii) BA 7 and 37. All cluster‐level connections were significant during the homophone and synonym judgments.

As the clustering patterns were different across the three tasks, we first manually reset the clusters in the phonological and semantic conditions so that they followed the clustering pattern in the orthographic condition. The justification is that both BA 9 and 46, as well as BA 7 and 37, were always clustered together across tasks, while the grouping of BA 44, 47, 21, and 6 varied. With this grouping, significant connectivity differences (after FDR correction) were found (i) between the cluster with BA 9 and 46 and the cluster with BA 44, 47, 21, and 6 (*F*(4,27) = 4.20, *p* = .0285); (ii) within the cluster with BA 44, 47, 21, and 6 (*F*(4,27) = 4.15, *p* = .0285); and (iii) within the cluster with BA 7 and 37 (F(2,29) = 4.64, *p* = .0357). At the connection level, only the connectivity between (i) BA 44 and 6 and (ii) BA 9 and 46 was significantly different across the three tasks after FDR correction (BA 44 and 6: *F*(2,29) = 9.68, *p* = .0169; BA 9 and 46: *F*(2,29) = 7.21, *p* = .0402). No ROI‐level task‐modulated connectivity difference survived FDR correction.

## 4 DISCUSSION

To the best of our knowledge, this is the first study that examined the brain activation and connectivity for Chinese reading during orthographic, phonological, and semantic processing using a within‐subject design. There were two main findings from our results. First, the three processes were supported by a left‐lateralized task‐common network, which included the left dorsolateral prefrontal cortex, FFG, and bilateral medial frontal gyri. Second, orthographic processing recruited a specialized network that is distinct from phonological and semantic processing, while the phonological and semantic networks were highly similar. Differences across tasks were mainly exhibited in the form of differences in activation strength and connectivity patterns. Orthographic processing was dominant in the superior portion of the left dorsolateral frontal region (BA 9/46), left SPL (BA 7), and bilateral FFG (BA 37). Semantics was dominant in areas located in the left pars triangularis (BA 45), pars orbitalis (BA 47), and MTG (BA 21). Phonological processing was dominant in areas found in the left dorsal precentral gyrus (BA 6). In the functional connectivity analysis, (i) BA 9 and 46 and (ii) BA 7 and 37 were consistently clustered into the same functional unit across the three tasks, while BA 6, 21, 44, and 47 exhibited a more varied pattern of grouping. Connectivity within the cluster with BA 7 and 37 was stronger during orthographic processing, while that within the cluster with BA 6, 21, 44, and 47 was stronger during phonological and semantic processing.

In the following sections, we discuss the implications of the results for neural networks associated with the reading of Chinese characters, with reference to the cognitive demands for processing each constituent of a Chinese character.

### 4.1 Specialized networks for orthographic, phonological, and semantic processing

#### 4.1.1 Orthographic processing

Within‐subject ANOVA, ROI and connectivity analyses revealed that there were specific regions and connectivity patterns for the three networks. Stronger activation levels in the left mid‐FFG during orthographic processing lends support to the assertion that the left mid‐fusiform area is a primary site for, if not specialized in, word form processing. Activations in the right FFG, left MFG (BA 9), and left SPL (BA 7) were also significantly stronger during orthographic processing. Previous studies discovered a developmental increase in activation in the left SPL, precuneus, MFG, bilateral MOG, and FFG during Chinese reading (Cao et al., 2010; Cao et al., 2015). Both the involvement of the right MOG and FFG have been suggested to be responsible for the visual analysis of the more complex and pictorial nature of Chinese characters (Cao et al., 2015; Guo & Burgund, 2010). It should be noted that activation of the FFG remained significant during phonological and semantic processing after the subtraction of activation during font‐size judgment. This implies that the mid‐fusiform area is involved in the finer visuo‐orthographic analysis of characters and not the recognition of the general outline of characters, and may be responsible for the integration of visual, phonological and semantic information (Qin et al., 2021).

The left SPL and LMFG have been hypothesized to be a frontal–parietal network that contributes to spatial working memory (Curtis, 2006; Ikkai & Curtis, 2011). Together with the FFG, these three regions exhibited significant connectivity during the orthography task. It can be inferred that orthographic processing in Chinese requires the recognition of the overall word form and the retention of the fine visuospatial details of stroke patterns. The concurrent activation of the FFG, and LMFG in the three tasks, together with the stronger connectivity between BA 7 and 37 during orthographic processing, suggests that orthographic processing is a nontrivial component in Chinese reading and requires cognitive support from an independent visuospatial network.

#### 4.1.2 Phonological processing

Significantly stronger activation during phonological processing was found in the left precentral gyrus (BA 6) and IFG (BA 44). Our results were consistent with most of the previous studies and meta‐analyses (Chen et al., 2002; Kuo et al., 2004; Siok et al., 2003; Siok et al., 2020; Tan et al., 2005; Wu et al., 2012). BA 44, being part of Broca's area, is a well‐documented region for speech sound processing, such as speech rehearsal (Price, 2012) and syllable counting (Poldrack et al., 1999). Our results reflected task requirements that the participants explicitly retrieve the pronunciation of the characters and the syllabic nature of Chinese characters.

Unlike some previous studies, we failed to find reliable evidence for the involvement of the STG/S. This region is in the proximity of the auditory cortex and has been identified with auditory processing, such as the analysis of speech and nonspeech sounds (Binder et al., 1996) and rapid frequency transitions (Poldrack et al., 2001). Reliance on the IFG and not the STS/G for phonological processing can be ascribed to the principles of phonological decoding: GPC does not exist with Chinese characters, so pronunciations must be retrieved through addressed phonology. In fact, Chen et al. (2002) found both IFG and STG activation in the pinyin > character contrast but only IFG activation in the character > pinyin contrast. Cao et al. (2010) and Cao et al. (2015) also found a developmental decrease in the activation of the left and right STG, respectively, which was attributed to reduced phonological representation among adults. With the absence of GPC and weak phonological representation of Chinese characters, it is not surprising that the STG/S does not constitute a part of the phonological processing circuit.

#### 4.1.3 Semantic processing

Semantic processing was found to uniquely utilize the pars triangularis and pars orbitalis in the left IFG (BA 45/47) and MTG (BA 21). Together with phonological processing, the activation level in the pars opercularis (BA 44) was also significantly stronger than that during orthographic processing. Consistent with both alphabetic and Chinese studies, these regions were commonly reported to be at the center of semantic retrieval, representation and comprehension (Bookheimer, 2002; Booth et al., 2006; Chee et al., 2001; Poldrack et al., 1999). With the exception of the task‐common regions of the LMFG and SPL, we did not find other peculiarities of the Chinese semantic network.

### 4.2 Functional segregation of the left lateral frontal cortex and the role of the LMFG

The ROI analysis revealed that the LMFG (BA 9/46) is specialized for orthographic processing in Chinese. The connectivity analysis further showed that BA 9 and 46 formed a functional unit that was consistent across tasks. In contrast, although BA 6, 44 and 47 are specialized for phonological and semantic processing, they were not consistently grouped together. We can, therefore, separate the left lateral frontal cortex into an orthographic network (LMFG) and a phonosemantic network (BA 6/44/47). During orthographic processing, we did not find connections between the visuospatial cluster (BA 7/37) and the phonosemantic cluster (BA 6/21/44/47). Instead, relatively strong connections were found between BA7 and BA9, BA9 and BA46, and BA46 and BA44/47 (Figure 4a and Table S4). The strong connectivity between the SPL and LMFG is consistent with the proposal that the LMFG is a projection from the SPL that is responsible for spatial working memory (Kravitz et al., 2011). Together with its domain‐general functions, such as working memory, executive control and attention, we hypothesize that the LMFG performs a major function in visuospatial analysis and acts as a platform for integrating visual and language functions, that is, intergrating the information from the visuospatial network and phonosemantic network. This hypothesis would explain the activation of the LMFG in various Chinese reading tasks, such as phonological (e.g., Kuo et al., 2004; Siok et al., 2003; Tan, Feng, et al., 2001) and semantic (e.g., Booth et al., 2006; Chee et al., 2000; Ding et al., 2003; Siok et al., 2004; Tan, Liu, et al., 2001; Wu et al., 2012) judgments. In the orthographic task, the LMFG itself performed its visuospatial analysis function, which led to a higher level of activation. Our results suggest that the LMFG is both an orthographic processing center and an interface between the visuospatial information and the phonosemantic networks.

### 4.3 Implications for the neural networks associated with Chinese reading

The whole‐brain activation patterns, ROI activation levels and ROI‐to‐ROI connectivity patterns suggest that orthographic processing vastly differs between phonological and semantic processing in Chinese. A dedicated network, which includes the left FFG, SPL, and MFG, is specialized for visuo‐orthographic processing of Chinese characters. In contrast, there was a high similarity between the phonological and semantic networks. In the connectivity analysis, there was no obvious clustering of BA 6, 21, 44, and 47 and the within‐cluster connections of these four regions were higher during phonological and semantic processing, implying that they were all involved in phonological and semantic processing. Additionally, in the ROI analysis, with the exception of the left precentral gyrus, stronger phonological processing than orthographic processing always implied stronger semantic processing. The activation pattern during phonological processing resembled that during semantic processing, although semantic processing elicited stronger activation of the left pars triangularis, pars orbitalis, and MTG. The close parallel between the phonology and semantic networks, with further specialization for semantic processing, suggests that phonological processing is always present when meaning is accessed, but the reverse is not true (i.e., semantic processing is not always performed when sounds are accessed). This pattern echoes the findings of behavioral studies that phonological activation is obligatory in Chinese reading (Spinks et al., 2000) while semantic processing is not.

In Chinese, most characters map to only one pronunciation but have many meanings, and many characters share the same pronunciation. Thus, even though phonology is activated early during character identification (Tan et al., 1995), it does not mediate access to meaning in Chinese. Therefore, character meaning is difficult to determine without context. This asymmetry in mapping is termed the determinacy principle (Tan & Perfetti, 1998). During the phonological task, the participants were not forced to select a particular meaning while it was required in the synonym judgment task. As a result, we failed to identify semantics‐specific regions during the phonological task, but extra resources from the semantic processing centers were recruited during the semantic task on top of the activation of the automatic phonological network. This explains why the semantic network contains all the major regions recruited during phonological processing.

In general, our findings reveal that orthographic processing in Chinese utilizes the bilateral FFG, left MFG and left SPL, whereas phonological and semantic processing recruits the left IFG (Broca's area) but not the left superior temporal region (Wernicke's area). These peculiarities should be interpreted with reference to the cognitive demand that the Chinese writing system imposes. In alphabetic languages, an indirect route by applying GPC is possible. The explicit decoding of graphemes into speech sound is closer to the auditory processing of language. Hence, the speech processing center (Wernicke's area) is involved in reading alphabetic languages. In Chinese, due to the absence of GPC, the phonology of a character is retrieved after visual form analysis. This phonological retrieval is believed to be done by the frontal regions. We do not claim that auditory processing does not play any role in Chinese. In fact, Broca's area was strongly activated during the phonological task, and a trend of stronger activation was exhibited in the STG during the phonological and semantic task than during the purely visual orthographic task (Table 4 and Figure 3). However, the mapping principle in Chinese weakens the role of auditory processing and rendered activation in the STG nonsignificant. In fact, a meta‐analysis of fMRI studies on dyslexic readers reported that only dyslexic readers of shallow orthographies demonstrate underactivation in the temporoparietal region (including STG/S), while dyslexic readers of deep orthographies do not (Martin et al., 2016). This lends support to the hypothesis that reading, or word decoding, engages the STG/S to different extents depending on the print‐to‐sound mapping principle.

Similarly, the stronger activation in the LMFG (BA 9/46) and SPL and their connectivity during the orthographic task should be interpreted with reference to the high visuospatial cognitive demand during Chinese reading rather than as a fundamentally different network from the occipitotemporal network for word form processing. Chinese characters are made up of basic stroke patterns that are assembled to form components, which are in turn organized into a square shape to form characters. Unlike alphabetic languages, in which a small set of letters (e.g., only 26 in English) are arranged linearly into words, there are approximately 560 components in Chinese (State Language Commission, 1998), and these components are packed into a two‐dimensional space. The distinction among components can be minute, such as 已 versus 己 (the 乚 stroke starts above the left end of the lower horizontal stroke in the first character, but it just touches the left end of the lower horizontal stroke in the second character) and 未 versus 末 (the upper horizontal stroke is shorter in the first character but longer in the second character). Moreover, different characters can be constructed from the same components but with different spatial arrangements, such as 棘 versus 棗 (the two 朿 's are arranged horizontally or vertically). These features tax resources for both visual perceptual skills (comparing the relative position and length of strokes) and spatial perceptual skills (comparing the relative position of components). Therefore, the right FFG is recruited for extra effort required for the visual analysis. In addition to recruiting the “what” ventral visual pathway from the visual areas (I/MOG) to the FFG for component identification, Chinese character recognition requires the “where” pathway (Mishkin et al., 1983; Ungerleider & Haxby, 1994) for the spatial analysis of components. Hence, the FFG and SPL formed a core functional unit supporting the visuospatial analysis of the characters. The brain network involved in processing a writing script is dependent on its linguistics features.

Our results highlight the importance of investigating the constituent components of reading separately. As reading is an umbrella term that encompasses more specific cognitive processes, it would be misleading to use a single reading task to represent the reading network of a language as a whole.

As a concluding remark, although our results appear to suggest that the network of Chinese reading differs from that of reading alphabetic languages, it should be stressed that this study did not include direct cross‐language comparisons, so it would be premature to draw conclusions. To determine whether Chinese reading engages a universal brain network, future studies should compare different orthographies. However, to avoid conflating the constituent reading processes, the orthographic, phonological, and semantic processing components should be investigated simultaneously to fully elucidate the reading profile of the studied languages.

## 5 CONCLUSION

We conclude that Chinese reading utilizes two major networks: the visuospatial network, which includes the left FFG, SPL, and MFG, to handle visuo‐orthographic information and the phonosemantic network, which includes the left precentral gyrus, IFG, and MTG, to retrieve and process phonological and semantic information. We propose that the neural mechanisms underlying Chinese reading correspond to the cognitive demands imposed by its peculiar visual and print‐to‐sound mapping features. With its capacity in visuospatial working memory, the LMFG acts as an interface between the posterior visuospatial network and the frontal phonosemantic network, supporting the processing of visually demanding Chinese scripts. Our study demonstrates the importance of considering the constituents of a writing system when examining its reading networks.

## Supporting information


**TABLE S1** MNI coordinates of the peaks found in the component judgment > line judgment contrast. The *p* values are uncorrected. The clusters survived a statistical significance of *p* < .05 with FWE correction. k: Cluster size. BA: Brodmann area.

**TABLE S2** MNI coordinates of the peaks found in the homophone judgment > font size judgment contrast. The *p* values are uncorrected. The clusters survived a statistical significance of *p* < .05 with FWE correction. k: Cluster size. BA: Brodmann area.

**TABLE S3** MNI coordinates of the peaks found in the synonym judgment > font size judgment contrast. The *p* values are uncorrected. The clusters survived a statistical significance of *p* < .05 with FWE correction. k: Cluster size. BA: Brodmann area.

**TABLE S4** Functional connectivity among ROIs. For component judgment, homophone judgment, synonym judgment and the between‐task contrasts, all reported clusters survived a *p*‐value threshold of .05 after FDR correction, while connections within the reported clusters may not. The between‐task contrast at connection‐level also survived a *p*‐value threshold of .05 after FDR correction.

## Data Availability

Data are available upon request from the corresponding author.
