# Supplementary material for: Functional connectivity during orthographic, phonological, and semantic processing of Chinese characters identifies distinct visuospatial and phonosemantic networks
Source: Hum Brain Mapp. 2022 Sep 12;43(16):5066–80. doi: 10.1002/hbm.26075 (PMC9582368; doi:10.1002/hbm.26075)
Supplement: Supplementary file 1 — TABLE S1 MNI coordinates of the peaks found in the component judgment > line judgment contrast. The p values are uncorrected. The clusters survived a statistical significance of p < .05 with FWE correction. k: Cluster size. BA: Brodmann area. [file HBM-43-5066-s004.docx]

| Regions | | k | BA | x | y | z | peak t | p |
| --- | --- | --- | --- | --- | --- | --- | --- | --- |
| Left | Superior parietal lobule | 2162 | 7 | -28 | -54 | 38 | 13.14 | 2.81E-14 |
|  | Precuneus |  | 7 | -10 | -72 | 40 | 4.75 | 2.33E-05 |
|  | Middle / inferior frontal gyrus | 8427 | 46 | -40 | 26 | 18 | 12.55 | 9.09E-14 |
|  |  |  | 46 | -40 | 34 | 12 | 9.66 | 5.07E-11 |
|  |  |  | 46 | -42 | 40 | 4 | 8.76 | 4.58E-10 |
|  |  |  | 46 | -46 | 44 | -2 | 7.33 | 1.81E-08 |
|  |  |  | 44 | -40 | 18 | 22 | 11.49 | 8.23E-13 |
|  |  |  | 6 | -28 | -2 | 56 | 9.39 | 9.64E-11 |
|  |  |  | 8 | -40 | 10 | 36 | 9.19 | 1.56E-10 |
|  | Inferior temporal gyrus / fusiform gyrus | 3858 | 37 | -36 | -38 | -28 | 12.42 | 1.17E-13 |
|  |  |  | 37 | -54 | -52 | -8 | 5.21 | 6.45E-06 |
|  |  |  | 20 | -50 | -34 | -26 | 9.25 | 1.37E-10 |
|  | Cerebellum | `` | - | -44 | -58 | -24 | 11.93 | 3.24E-13 |
|  |  |  | - | -40 | -44 | -32 | 10.97 | 2.52E-12 |
|  |  |  | - | -40 | -76 | -22 | 8.4 | 1.14E-09 |
|  | Thalamus | 220 | - | -26 | -24 | 0 | 6.25 | 3.44E-07 |
|  | Hippocampus | `` | 54 | -32 | -16 | -16 | 5.02 | 1.09E-05 |
|  |  |  | - | -30 | -24 | -8 | 4.88 | 1.65E-05 |
|  | Middle occipital gyrus | 186 | - | -32 | -80 | 18 | 4.95 | 1.33E-05 |
| Right | Fusiform gyrus / Parahippocampal gyrus | 1399 | 37 | 38 | -70 | -20 | 6.47 | 1.89E-07 |
|  |  |  | - | 34 | -66 | -46 | 6.16 | 4.45E-07 |
|  |  |  | 36 | 38 | -24 | -24 | 4.29 | 8.52E-05 |
|  | Occipital fusiform gyrus | `` | 19 | 32 | -86 | -16 | 5.5 | 2.88E-06 |
|  | Cerebellum | `` | - | 38 | -68 | -30 | 6.78 | 8.07E-08 |
|  |  |  | - | 38 | -44 | -30 | 6.72 | 9.53E-08 |
|  | Middle / inferior frontal gyrus | 970 | 44 | 40 | 14 | 30 | 6.51 | 1.69E-07 |
|  |  |  | 9 | 44 | 26 | 24 | 4.97 | 1.27E-05 |
|  | Insula | `` | 13 | 30 | 28 | 2 | 5.63 | 1.95E-06 |
|  | Precentral gyrus | 440 | 6 | 40 | -2 | 52 | 6.37 | 2.52E-07 |
| Medial | Cerebellum | 762 | - | 6 | -74 | -40 | 7.01 | 4.32E-08 |
|  |  |  | - | -4 | -72 | -36 | 5.59 | 2.19E-06 |

TableS1. MNI coordinates of the peaks found in the component judgement > line judgement contrast. The p values are uncorrected. The clusters survived a statistical significance of p < 0.05 with FWE correction. k: Cluster size. BA: Brodmann area.
