# Supplementary material for: Functional connectivity during orthographic, phonological, and semantic processing of Chinese characters identifies distinct visuospatial and phonosemantic networks
Source: Hum Brain Mapp. 2022 Sep 12;43(16):5066–80. doi: 10.1002/hbm.26075 (PMC9582368; doi:10.1002/hbm.26075)
Supplement: Supplementary file 2 — TABLE S2 MNI coordinates of the peaks found in the homophone judgment > font size judgment contrast. The p values are uncorrected. The clusters survived a statistical significance of p < .05 with FWE correction. k: Cluster size. BA: Brodmann area. [file HBM-43-5066-s002.docx]

| Regions | | k | BA | x | y | z | peak t | p |
| --- | --- | --- | --- | --- | --- | --- | --- | --- |
| Left | Middle / inferior frontal gyrus | 8321 | 46 | -40 | 26 | 20 | 10.95 | 2.64E-12 |
|  |  |  | 8 | -40 | 10 | 28 | 10.82 | 3.53E-12 |
|  |  |  | 44 | -40 | 18 | 24 | 9.84 | 3.34E-11 |
|  |  |  | 45 | -44 | 36 | 4 | 7.51 | 1.12E-08 |
|  |  |  | 47 | -34 | 28 | 0 | 6.95 | 5.05E-08 |
|  | Precentral gyrus | `` | 6 | -48 | 0 | 54 | 8.55 | 7.78E-10 |
|  | Fusiform gyrus / inferior temporal gyrus | 1422 | 37 | -42 | -38 | -28 | 5.79 | 1.24E-06 |
|  |  |  | 37 | -46 | -52 | -20 | 5.64 | 1.90E-06 |
|  |  |  | 37 | -58 | -56 | -4 | 4.21 | 1.06E-04 |
|  | Middle temporal gyrus | `` | 21 | -44 | -40 | 2 | 3.97 | 2.06E-04 |
|  | Cerebellum | `` | - | -44 | -48 | -34 | 5.26 | 5.59E-06 |
|  |  |  | - | -48 | -64 | -24 | 5.03 | 1.07E-05 |
|  |  |  | - | -34 | -40 | -34 | 4.04 | 1.73E-04 |
|  | Occipital fusiform gyrus | 532 | 18 | -26 | -86 | -14 | 7.25 | 2.25E-08 |
|  | Cerebellum | `` | - | -30 | -86 | -26 | 4.52 | 4.48E-05 |
|  |  |  | - | -38 | -80 | -28 | 3.78 | 3.52E-04 |
|  | Superior parietal lobule | 413 | 7 | -28 | -54 | 34 | 7.14 | 3.03E-08 |
| Right | Inferior frontal gyrus | 725 | 47 | 34 | 40 | -10 | 4.67 | 2.95E-05 |
|  | Insula | `` | 13 | 32 | 26 | 0 | 4.82 | 1.95E-05 |
|  | Cerebellum | 519 | - | 6 | -72 | -34 | 6.26 | 3.41E-07 |
|  |  |  | - | 8 | -72 | -44 | 5.21 | 6.47E-06 |
|  | Caudate | 432 | - | 22 | 4 | 20 | 6.48 | 1.86E-07 |
|  |  |  | 48 | 16 | -14 | 20 | 5.92 | 8.67E-07 |
|  | Inferior occipital gyrus | 223 | 17 | 8 | -70 | 6 | 4.86 | 1.71E-05 |
|  |  |  | 18 | 0 | -80 | 0 | 3.71 | 4.22E-04 |
|  |  |  | 18 | 20 | -66 | 4 | 3.54 | 6.58E-04 |
| Medial | Supplementary motor area | 1753 | 8 | 10 | 22 | 48 | 6.37 | 2.49E-07 |
|  |  |  | 6 | -4 | 2 | 62 | 6.36 | 2.57E-07 |
|  | Superior frontal gyrus | `` | 8 | -2 | 30 | 46 | 6.19 | 4.12E-07 |

TableS2. MNI coordinates of the peaks found in the homophone judgement > font size judgement contrast. The p values are uncorrected. The clusters survived a statistical significance of p < 0.05 with FWE correction. k: Cluster size. BA: Brodmann area.
