# Supplementary material for: Functional connectivity during orthographic, phonological, and semantic processing of Chinese characters identifies distinct visuospatial and phonosemantic networks
Source: Hum Brain Mapp. 2022 Sep 12;43(16):5066–80. doi: 10.1002/hbm.26075 (PMC9582368; doi:10.1002/hbm.26075)
Supplement: Supplementary file 3 — TABLE S3 MNI coordinates of the peaks found in the synonym judgment > font size judgment contrast. The p values are uncorrected. The clusters survived a statistical significance of p < .05 with FWE correction. k: Cluster size. BA: Brodmann area. [file HBM-43-5066-s003.docx]

| Regions | | k | BA | x | y | z | peak t | p |
| --- | --- | --- | --- | --- | --- | --- | --- | --- |
| Left | Middle / Inferior frontal gyrus | 6970 | 44 | -48 | 20 | 26 | 14.57 | 2.11E-15 |
|  |  |  | 45 | -48 | 32 | 10 | 8.96 | 2.78E-10 |
|  |  |  | 47 | -44 | 32 | -4 | 10.29 | 1.17E-11 |
|  |  |  | 47 | -48 | 24 | -8 | 7.56 | 9.81E-09 |
|  | Insula | `` | 13 | -26 | 26 | -6 | 10.08 | 1.90E-11 |
|  | Middle frontal gyrus / precentral gyrus | `` | 8 | -40 | 12 | 28 | 10.87 | 3.13E-12 |
|  |  |  | 6 | -50 | 8 | 48 | 7.11 | 3.29E-08 |
|  |  |  | 6 | -38 | -4 | 64 | 6.03 | 6.33E-07 |
|  | Medial superior frontal gyrus / | 1397 | 8 | -6 | 22 | 50 | 8.05 | 2.73E-09 |
|  | supplementary motor area |  | 8 | 0 | 28 | 46 | 7.66 | 7.71E-09 |
|  |  |  | 6 | -2 | 14 | 56 | 7.33 | 1.80E-08 |
|  | Inferior temporal gyrus / fusiform gyrus | 1772 | 37 | -52 | -42 | -24 | 6.78 | 8.06E-08 |
|  |  |  | 37 | -44 | -50 | -22 | 5.83 | 1.12E-06 |
|  |  |  | 37 | -60 | -58 | -8 | 4.17 | 1.18E-04 |
|  | Middle temporal gyrus | `` | 21 | -50 | -40 | -6 | 6.34 | 2.70E-07 |
|  | Superior parietal lobule | 261 | 7 | -28 | -58 | 42 | 5.54 | 2.55E-06 |
|  | Inferior occipital gyrus | 493 | 18 | -30 | -90 | -10 | 5.48 | 3.02E-06 |
|  |  |  | 18 | -30 | -88 | -20 | 5.17 | 7.22E-06 |
|  |  |  | 18 | -32 | -88 | 2 | 4.40 | 6.28E-05 |
|  | Cerebellum | `` | - | -40 | -76 | -26 | 3.63 | 5.19E-04 |
| Right | Insula | 634 | 13 | 30 | 24 | -4 | 7.40 | 1.53E-08 |
|  | Inferior frontal gyrus | `` | 45 | 30 | 30 | 4 | 6.24 | 3.58E-07 |
|  |  |  | 47 | 34 | 32 | -6 | 5.32 | 4.75E-06 |
|  | Cerebellum | 299 | - | 12 | -74 | -44 | 6.72 | 9.47E-08 |

TableS3. MNI coordinates of the peaks found in the synonym judgement > font size judgement contrast. The p values are uncorrected. The clusters survived a statistical significance of p < 0.05 with FWE correction. k: Cluster size. BA: Brodmann area.
