# Supplementary material for: Functional connectivity during orthographic, phonological, and semantic processing of Chinese characters identifies distinct visuospatial and phonosemantic networks
Source: Hum Brain Mapp. 2022 Sep 12;43(16):5066–80. doi: 10.1002/hbm.26075 (PMC9582368; doi:10.1002/hbm.26075)
Supplement: Supplementary file 4 — TABLE S4 Functional connectivity among ROIs. For component judgment, homophone judgment, synonym judgment and the between‐task contrasts, all reported clusters survived a p‐value threshold of .05 after FDR correction, while connections within the reported clusters may not. The between‐task contrast at connection‐level also survived a p‐value threshold of .05 after FDR correction. [file HBM-43-5066-s001.docx]

Table S4. Functional connectivity among ROIs. For component judgement, homophone judgement, synonym judgement and the between-task contrasts, all reported clusters survived a p-value threshold of 0.05 after FDR correction, while connections within the reported clusters may not. The between-task contrast at connection-level also survived a p-value threshold of 0.05 after FDR correction.

| **Analysis Unit** | **Statistic** | **p-uncorrected** | **p-FDR** |
| --- | --- | --- | --- |
| **Component judgement** | | | |
| *Within-cluster connection: {BA9, 46}* | F(1,30) = 120.24 | < 0.0000005 | < 0.0000005 |
| BA9-BA46 | T(30) = 10.97 | < 0.0000005 | < 0.0000005 |
| *Between-cluster connection: {BA9, 46} - {BA 6, 21, 44, 47}* | F(2,29) = 81.25 | < 0.0000005 | < 0.0000005 |
| BA46-BA47 | T(30) = 10.00 | < 0.0000005 | < 0.0000005 |
| BA46-BA44 | T(30) = 8.45 | < 0.0000005 | < 0.0000005 |
| BA9-BA44 | T(30) = 5.86 | 0.000002 | 0.000005 |
| BA9-BA6 | T(30) = 5.26 | 0.000011 | 0.00002 |
| BA46-BA21 | T(30) = 5.21 | 0.000013 | 0.000022 |
| BA46-BA6 | T(30) = 5.12 | 0.000016 | 0.000023 |
| BA9-BA47 | T(30) = 4.90 | 0.000031 | 0.000043 |
| *Between-cluster connection: {BA9, 46} - {BA 7, 37}* | F(2,29) = 37.06 | < 0.0000005 | < 0.0000005 |
| BA9-BA7 | T(30) = 9.53 | < 0.0000005 | < 0.0000005 |
| BA46-BA7 | T(30) = 4.35 | 0.000143 | 0.000167 |
| BA9-BA37 | T(30) = 3.26 | 0.002749 | 0.003207 |
| *Within-cluster connection: {BA 6, 21, 44, 47}* | F(2,29) = 31.95 | < 0.0000005 | < 0.0000005 |
| BA44-BA47 | T(30) = 7.08 | < 0.0000005 | < 0.0000005 |
| BA44-BA6 | T(30) = 3.96 | 0.000432 | 0.000756 |
| BA47-BA21 | T(30) = 3.84 | 0.000594 | 0.001039 |
| BA44-BA21 | T(30) = 3.38 | 0.002043 | 0.00286 |
| BA21-BA6 | T(30) = 3.34 | 0.00224 | 0.003921 |
| BA47-BA6 | T(30) = 2.18 | 0.037384 | 0.043615 |
| *Within-cluster connection: {BA 7, 37}* | F(1,30) = 26.40 | 0.000016 | 0.000019 |
| BA7-BA37 | T(30) = 5.14 | 0.000016 | 0.000055 |
| **Homophone judgement** |  |  |  |
| *Between-cluster connection: {BA9, 44, 46} - {BA 6, 47}* | F(2,29) = 125.23 | < 0.0000005 | < 0.0000005 |
| BA46-BA47 | T(30) = 10.79 | < 0.0000005 | < 0.0000005 |
| BA9-BA6 | T(30) = 8.94 | < 0.0000005 | < 0.0000005 |
| BA44-BA6 | T(30) = 8.67 | < 0.0000005 | < 0.0000005 |
| BA46-BA6 | T(30) = 8.06 | < 0.0000005 | < 0.0000005 |
| BA44-BA47 | T(30) = 7.14 | < 0.0000005 | < 0.0000005 |
| BA9-BA47 | T(30) = 5.43 | 0.000007 | 0.000012 |
| *Within-cluster connection: {BA9, 44, 46}* | F(2,29) = 87.48 | < 0.0000005 | < 0.0000005 |
| BA9-BA46 | T(30) = 11.88 | < 0.0000005 | < 0.0000005 |
| BA9-BA44 | T(30) = 9.94 | < 0.0000005 | < 0.0000005 |
| BA46-BA44 | T(30) = 9.19 | < 0.0000005 | < 0.0000005 |
| *Between-cluster connection: {BA 6, 47} - {BA 7, 21, 37}* | F(2,29) = 24.46 | 0.000001 | 0.000001 |
| BA6-BA21 | T(30) = 5.53 | 0.000005 | 0.000009 |
| BA47-BA21 | T(30) = 3.71 | 0.000838 | 0.001467 |
| BA6-BA37 | T(30) = 3.16 | 0.003547 | 0.004138 |
| BA47-BA7 | T(30) = 2.78 | 0.009396 | 0.010962 |
| BA6-BA7 | T(30) = 2.61 | 0.013914 | 0.013914 |
| *Between-cluster connection: {BA9, 44, 46} - {BA 7, 21, 37}* | F(2,29) = 19.62 | 0.000004 | 0.000006 |
| BA46-BA21 | T(30) = 4.49 | 0.000097 | 0.000135 |
| BA9-BA37 | T(30) = 4.20 | 0.000218 | 0.000305 |
| BA46-BA7 | T(30) = 3.90 | 0.000498 | 0.000581 |
| BA9-BA7 | T(30) = 3.85 | 0.000581 | 0.000677 |
| BA44-BA21 | T(30) = 3.86 | 0.000566 | 0.000792 |
| BA9-BA21 | T(30) = 3.42 | 0.001811 | 0.001811 |
| BA46-BA37 | T(30) = 2.80 | 0.008926 | 0.008926 |
| *Within-cluster connection: {BA 6, 47}* | F(1,30) = 11.00 | 0.002393 | 0.002871 |
| BA47-BA6 | T(30) = 3.32 | 0.002393 | 0.00335 |
| *Within-cluster connection: {BA 7, 21, 37}* | F(2,29) = 3.47 | 0.044436 | 0.044436 |
| BA7-BA37 | T(30) = 2.81 | 0.008597 | 0.016442 |
| **Synonym judgement** |  |  |  |
| *Within-cluster connection: {BA9, 44, 46, 47}* | F(2,29) = 117.63 | < 0.0000005 | < 0.0000005 |
| BA46-BA47 | T(30) = 12.55 | < 0.0000005 | < 0.0000005 |
| BA9-BA46 | T(30) = 12.28 | < 0.0000005 | < 0.0000005 |
| BA46-BA44 | T(30) = 9.91 | < 0.0000005 | < 0.0000005 |
| BA44-BA47 | T(30) = 9.64 | < 0.0000005 | < 0.0000005 |
| BA9-BA44 | T(30) = 9.44 | < 0.0000005 | < 0.0000005 |
| BA9-BA47 | T(30) = 7.80 | < 0.0000005 | < 0.0000005 |
| *Between-cluster connection: {BA6, 21} - {BA9, 44, 46, 47}* | F(2,29) = 91.95 | < 0.0000005 | < 0.0000005 |
| BA44-BA6 | T(30) = 8.07 | < 0.0000005 | < 0.0000005 |
| BA9-BA6 | T(30) = 7.87 | < 0.0000005 | < 0.0000005 |
| BA46-BA21 | T(30) = 6.46 | < 0.0000005 | 0.000001 |
| BA44-BA21 | T(30) = 6.23 | 0.000001 | 0.000001 |
| BA47-BA21 | T(30) = 6.01 | 0.000001 | 0.000002 |
| BA46-BA6 | T(30) = 5.79 | 0.000003 | 0.000004 |
| BA47-BA6 | T(30) = 5.34 | 0.000009 | 0.000012 |
| BA9-BA21 | T(30) = 4.59 | 0.000073 | 0.000085 |
| *Between-cluster connection: {BA7, 37} - {BA9, 44, 46, 47}* | F(2,29) = 14.13 | 0.000052 | 0.000104 |
| BA9-BA7 | T(30) = 5.89 | 0.000002 | 0.000003 |
| BA9-BA37 | T(30) = 3.28 | 0.002633 | 0.002633 |
| BA44-BA7 | T(30) = 3.24 | 0.002934 | 0.003423 |
| BA46-BA7 | T(30) = 3.18 | 0.003412 | 0.003981 |
| BA46-BA37 | T(30) = 2.20 | 0.035456 | 0.035456 |
| *Within-cluster connection: {BA6, 21}* | F(1,30) = 19.88 | 0.000107 | 0.00016 |
| BA6-BA21 | T(30) = 4.46 | 0.000107 | 0.000149 |
| *Within-cluster connection: {BA7, 37}* | F(1,30) = 16.05 | 0.000375 | 0.00045 |
| BA7-BA37 | T(30) = 4.01 | 0.000375 | 0.001313 |
| *Between-cluster connection: {BA7, 37} - {BA6, 21}* | F(2,29) = 7.28 | 0.002742 | 0.002742 |
| BA6-BA37 | T(30) = 3.10 | 0.004171 | 0.004866 |
| BA6-BA7 | T(30) = 2.85 | 0.007823 | 0.007823 |
| **Between-task connectivity difference** | | | |
| **cluster level** | | | |
| *Between-cluster: {BA9, 46} - {BA 6, 21, 44, 47}* | F(4,27) = 4.20 | 0.008982 | 0.028518 |
| BA9-BA6 | F(2,29) = 7.21 | 0.002874 | 0.020116 |
| BA46-BA47 | F(2,29) = 4.35 | 0.022225 | 0.101969 |
| BA46-BA44 | F(2,29) = 4.00 | 0.029134 | 0.101969 |
| BA9-BA47 | F(2,29) = 3.57 | 0.041077 | 0.143768 |
| *Within-cluster: {BA 6, 21, 44, 47}* | F(4,27) = 4.15 | 0.009506 | 0.028518 |
| BA44-BA6 | F(2,29) = 9.68 | 0.000604 | 0.004225 |
| *Within-cluster: {BA 7, 37}* | F(2,29) = 4.64 | 0.017834 | 0.035668 |
| BA7-BA37 | F(2,29) = 4.64 | 0.017834 | 0.124838 |
| **connection level** | | | |
| BA44-BA6 | F(2,29) = 9.68 | 0.000604 | 0.016899 |
| BA9-BA6 | F(2,29) = 7.21 | 0.002874 | 0.040233 |
